# Working from Home: Is Our Housing Ready?

**DOI:** 10.3390/ijerph18147329

**Published:** 2021-07-08

**Authors:** Teresa Cuerdo-Vilches, Miguel Ángel Navas-Martín, Ignacio Oteiza

**Affiliations:** 1 Instituto de Ciencias de la Construcción Eduardo Torroja, Consejo Superior de Investigaciones Científicas (IETcc-CSIC), 28033 Madrid, Spain; ioteiza@ietcc.csic.es; 2 Escuela Nacional de Sanidad, Instituto de Salud Carlos III (ISCIII), 28029 Madrid, Spain; manavas@isciii.es

**Keywords:** COVID-19, confinement, telework, comfort, home spaces, telework space adequacy index (TSAI), photo, narrative, mixed-method, remote work

## Abstract

The COVID-19 pandemic and the precautionary measures applied globally (lockdowns and curfews) have impacted homes, including work. Working from home (WFH) has emerged as a growing trend in the post-pandemic era. The research question was: *Are our homes ready for teleworking?* To respond, a national prospective mixed approach was launched for Spanish households during the spring 2020 lockdown, using two online questionnaires, one quantitative and the other qualitative. Through a survey, photographs, and narratives, the study evaluates the perceived adequacy of telework spaces and their specific characteristics, the availability of digital resources and the internet. A total of 1800 surveys and over 200 images and texts related to telework environments were obtained. The results suggest that the adequacy of these spaces was insufficient for more than a quarter of the homes. Also, strong relations between the perceived workspace adequacy and a social status or stability of homes were shown and validated, despite other sociodemographic features, the home composition or habitat were not related. Some other variables statistically significant were occupation regime, type and surface of dwellings; their indoor environmental quality; the availability of exclusive spaces for teleworking; quality of digital resources; and the specific space features. The analysis was completed with qualitative insights through photos and texts. Telework, lived in this context as an experiment, needs this reflection from an environmental, resource-availability, and ergonomic point of view.

## 1. Introduction

With the outbreak of the COVID-19 pandemic, confinement was stablished as a preventive and containment measure for public health at a global level, and more specifically, in European countries, such as Spain [1]. Confinement and social distance have had a psychological impact on the population [2], hitting the most disadvantaged in a more significant and general way [3]. As a consequence, it generates the worsening of the already unequal distribution of wealth [4].

Work activity has suffered very differently depending on the sector, the sociodemographic characteristics, the qualification, and the geographical area or the level of restriction imposed, among others [5]. One of the consequences at a global level has been the promotion of teleworking [6]. It supposed not only a work modality, but also a preventive measure against coronavirus contagion for the active population and their environments [7].

The terminological diversity that leads to the concepts “working from home (WFH)”, “remote working”, or “telework”, depending on the study, is striking. Apparently, the nuances of their use depend mainly on the type of worker and the place where the task is carried out, although they are inevitably related and sometimes even overlap [8].“Telework” purely refers to the work that is done in any place other than the usual. “Working from home”, refers to when the work is done partially or totally in the worker’s home [9]. Although the diversity of terms makes it difficult to locate the related bibliography, in this study, the term “telework” is mainly used (“tele-study” if the activity is related to students), although the terms were eventually used as synonyms.

A well-extended pre-pandemic attitude on the part of managers and employers has been to reject teleworking. Some of the reasons were a preference to follow traditional work models [10], as well as the implementation and improvement in the processes and information systems necessary to make teleworking operational within companies [11]. Some studies refer to the differences between the implementation of teleworking distinguishing between SMEs and large companies [12]. Digital transformation has become necessary in homes susceptible to teleworking [13]. However, this has led to the question of whether we are facing a new digital divide [14].

Undoubtedly, there is a trend in the occupational capacity to assume teleworking. Certain areas and professional sectors better support the remote work modality than others. Nevertheless, teleworking, being easily implemented, is still mainly rejected in some sectors, such as the Public Administration and other activities. Otherwise, it is traditionally more common among self-employed, more qualified people, and small companies [15].

This has led to qualify certain sociodemographic factors, such as gender, due to women being more likely to adopt this modality [8]. The closure of educational centers has been another measure taken due to the confinement. When possible, teleworking has also provided a national solution to relocate those professional tasks that could be undertaken remotely [16]. Many women have considered staying at home, to reconcile family and working life [17], at least, as long as the measure lasted. Comparatively, women have stayed the most at home [18], either due to increased pressure and job uncertainty, such as caring for minors and other dependents, or due to social conventions, as a general situation reported by United Nations [19]. This fact has potentially generated some frustration due to productivity and perceived satisfaction in the professional aspect [20].

Initially, teleworking contributed not only to people’s safety, but also to a drastic decrease in environmental pressure by reducing mobility [21]. However, the possible benefit on the family nucleus and the perception of the balance between home care and work activity was not so clear [22].

In Spain, confinement was addressed through the State of Alarm decree [23], three days after the World Health Organization officially declared the COVID-19 pandemic [24]. It lasted until 22 June 2020 [25]. This has conducted a leading role for housing. Homes responded unevenly in terms of resilience to the new needs of confined cohabitants [26,27,28], mainly in the first weeks.

Telework has been a minority labor modality especially in Spain, of recent adoption due to the pandemic. This fact has conditioned how households have assumed this way of working. Some questions arise around the preparation of homes to this end. Beyond a stable access to supplies such as energy or adequate internet bandwidth, the indoor environmental quality of these spaces, their habitability, comfort, or ergonomics are just some of the questions raised about workspaces. In addition, it is important to know what teleworkers are looking for in a workspace at home, and to what degree these preferences have been satisfied.

In Spain, before confinement, the proportion of people who teleworked habitually was 4.8%. During the lockdown, in the spring of 2020, this percentage increased to 9.9%, with a substantial change in the way of developing telework. After confinement, in the first quarter of 2021, it stood at 11.2% [29]. It has been as a result of the pandemic, when this way of working has notably irrupted, since traditionally this type of work was very limited.

In the case of Spain, a legal framework was established after the confinement situation to regulate working conditions for people who work remotely. Among the main measures, in addition to the provision of the necessary means and its voluntary nature, some incentives were added to favor teleworking in rural areas, as a measure to reverse the depopulation of mainly non-urban territories [30]. Likewise, in the second semester of 2020, teleworking was regulated in the Public Administrations, so that this way of working was organized and structured correctly, guaranteeing the operation and service to the general interests [31].

Regarding the support networks, during the pandemic there have been numerous service initiatives in local communities and Spanish neighborhoods. Some of them were carried out at the municipal policy level. For instance, in Bilbao, accommodation for homeless people was provided and citizen collaboration was also requested to identify people with basic difficulties, among other measures [32]. Other initiatives were carried out by neighborhood associations, for example, by the Neighborhood Associations in Madrid who held protests to request the opening of health centers in their area [33]. In Seville, the RAMUCA support network carried out different support actions, including help with shopping and psychological support [34].

According to the good practices guide, for the context of teleworking during the pandemic, it was recommended to create social networks that allow support for the tele-study in the neighborhood environment, with the help of trusted people to attend to school issues for young people. Caregiving support, for example by taking care shifts, was promoted to facilitate teleworking without interruptions, as well as generate communal spaces and community care. In addition, “time banks” were proposed to boost the exchange of time to carry out different domestic chores [35].

The aforementioned literature shows interesting aspects about telework, which directly affect the understanding of this type of work and its uneven implementation according to a multitude of factors to take into account, internal and external to the home. However, no study has been located that analyzes in detail the aspects of the workspace in the home from the housing and general quality point of view, taking into account in turn different aspects that intervene in its perception, as they may be environmental, spatial, ergonomic, functional, or resource aspects that affect the final assessment and quality of the same. Therefore, the main aim of this study is to analyze in depth the nature of these teleworking spaces in homes, and their adequacy, considering multiple factors, in the context of confinement. This means that, unlike normal circumstances, other variables such as the presence of minors in the home, or sharing the telework space, for example, have been also considered. The general research question has been: Are our homes ready to telework? This question needs to be answered bearing in mind the extreme situation of the lockdown of the whole household. As this phenomenon is considered unprecedented at a global level in terms of continuously teleworking at home, imposed by health and government institutions, and with the rest of the household present 24 h a day, the phenomenon was approached from a mixed perspective, allowing us to both collect relevant data by number of responses, as well as other insights from a qualitative perspective. This was done using photos, supported by contextualizing narrative from participants.

Quantitative and qualitative approaches allowed us to taxonomize this teleworking space and its degree of adaptation with respect to its users. A questionnaire also covered general aspects about the adaptation of the households and dwellings to the needs of confinement by COVID-19. These aspects were complemented with the request of a workspace photograph, three labels to characterize it, and five contextual questions about what was photographed, the intention of taking the photo, and the possible social contribution through open reflections. This qualitative analysis starts from a methodological adaptation of a technique called Photovoice [36,37,38,39], that tries to show reality through graphics and testimonials, in more detail and open to discovery. This also made possible to adapt it to the online form format, to make it reach confined persons as much as possible.

With the mixed approach, this study analyzed the phenomenon of teleworking in Spain, in general, within the home, under the context of confinement. More specifically, the main qualities of teleworking households were evaluated, as well as the characteristics that define whether a telework space at home is adequate or not, and other related aspects. A telework space adequacy index (TSAI) was also created, based on such features, and complemented with qualitative analysis. All those approaches allow for enriching, complementing, and qualifying the responses obtained in the questionnaire.

These approximations to the quality of teleworking spaces were evaluated taking into consideration both the households and dwellings characteristics, for the entire national territory.

## 2. Materials and Methods

To carry out this research, a mixed method was applied from two different approaches, quantitative and qualitative. The objective of applying the two approaches was based on complementing the views that each one of them contributed about what has been considered a complex phenomenon with dimensions of impact still unknown in many areas. This singular and extreme context of domestic confinement for reasons of SARS-CoV-2, could be more easily threshed, to know in a comprehensive way, the relationship of the cohabitants, how the confinement has been experienced, and what role it has played that unique space for meeting and safety, which has been the house.

This study received a favorable report from the Ethics Committee of the Spanish National Research Council (CSIC), with approval code number 057/2020.

### 2.1. Publicity of the Study and Search for Participants

To reach potential participants, given the added difficulty of confinement in terms of personal contact, the samples were taken through the internet, with independent self-completed forms. To this end, the study was publicized on multiple internet channels: e-mail, instant messaging Apps (such as Whatsapp^®^), institutional websites, social media, and mass media. A search and subsequent massive sending of emails through web scraping to reach all possible municipalities in Spain were also used for a greater potential recruitment.

The target was subjects older than 18 years, representatives of their homes, who remained the confinement in their usual homes. The two forms were completely anonymous, made on an online platform accessible from any device with an internet connection, each one from a different approach. Therefore, in both forms, initial questions about socio-demographic data were collected, in order to describe the participation in each part of the study.

Finally, both approaches were crossed to try to understand the phenomenon more broadly, building a joint discourse from all the information obtained.

In this contribution, emphasis was placed on everything related to telework or tele-study and the spaces and resources available in the participating homes.

### 2.2. Quantitative Study (Questionnaire)

The quantitative questionnaire was made up of 58 questions. They were grouped with diverse themes in relation to the homes and dwellings during confinement. Among daily habits and activities, questions were specifically asked related to telework and tele-study and the members of the household, as well as about the spaces and resources available to the home for their daily performance. From all these questions, the analysis of the responses was established, and the possible relationships among them, contrasted with the Chi-square method.

The analysis of the survey questions was carried out from different approaches. In the first place, a descriptive study was established by frequencies, for each variable, for which previous inconsistencies were eliminated and minority responses or categories that contributed little to the analysis were regrouped. The software used was SPSS (IBM Corp., Armonk, NY, USA), version 26.

Subsequently, crossovers were made between variables that apparently could have a certain relationship, to see if it was significant. For this, different hypotheses of bivariate independence were established, which were verified with different parameters, highlighting Pearson’s Chi-square, and other complementary ones, such as linear relationships.

Afterwards, the variable of adaptation to the telework space was chosen, to establish the relationships with the variables of household and dwelling, as well as the different aspects of these spaces valued by the participants, to see its incidence, establishing a Telework Space Adaptation Index (TSAI), as a ranking.

Some calculations were also included to validate the construct and this Telework Space Adaptation Index. Also, to prove and validate the relations among independent variables (home characteristics and telework space features), and the dependent one, the adequacy of telework space, adjusted binary-logistic regressions were carried out, and a Classification Tree, using SPSS version 26 statistical software.

### 2.3. Qualitative Study (Photos and Written Narratives)

The qualitative form requested information of a diverse nature, around certain areas of the house. After the initial sociodemographic information, and in relation to telework, the participant was required to take a photograph of the space where they carried out this activity. The objective was to complement the information of the quantitative questionnaire, describing graphically and verbally the perception of this space. For each photograph, three tags with keywords were requested to be able to categorize them. Finally, it was accompanied by the answers to five questions with a specific structure, which have their origin in a methodological adaptation of methods such as Photovoice [40,41] or photo-elicitation [33]. The objective of answering these questions is to be able to understand the purpose of capturing the photograph in this way and not in another, the intentionality, and what the author considered that he could contribute to other people. The questions asked for the photographs were as follows:

- what do you see in the image?
- what is happening in the image?
- why did you make this image?
- what does this image express about your life now, during confinement?
- what message could this image give to other people to improve their lives?

From the analysis of all these qualitative data, and because group sessions could not be initially counted as part of this Photovoice technique, the researchers were able to construct the discursive thread, to give greater robustness to the information obtained by the study and reach a greater understanding of the phenomenon under study.

To analyze the images, they were previously coded with alphanumeric codes that facilitated their referencing and assignment of attributes.

Once renamed, these were graphically analyzed, extracting information that was gathered into categories related to parts, elements, or characteristics that may affect the adequacy of workspaces. The categories arising from the observation of the images themselves could be completed with the information in the written texts provided by their authors, specifically those related to the questions: “What do you see in the image?” and “What is happening in the image?”.

After selecting the terms and concepts that would make up the final categorization, the verbatims (transcribed texts) related to these questions were analyzed to complete the information obtained. The qualitative analysis came to an end when the information saturation was reached, and therefore when the data sources could no longer contribute anything more to the discourse. In other words, data saturation is produced when no new information is generated [42].

For the textual analysis of the responses received, Content Analysis was used. For this, the verbatims and labels were coded to maintain the relationship with the photographs they described. The content analysis was done in different phases. First, the frequency of words was used, for which empty words were eliminated and those with the same root were grouped. Word clouds were generated to show this hierarchy of words. The software used was NVivo software (QSR International–Americas, Burlington, CT, USA) release 1.3.

Finally, a final categorization and a comprehensive analysis were reached by the researchers, which brought together all the stages of collecting results from data sources. Taking into account all the elements, terms, or concepts related to the phenomenon of housing in confinement, it also includes small insights without apparent relation to the issues exposed, so as to contribute to the understanding, qualification, or enrichment of what is revealed in the quantitative part of the study.

## 3. Results

Next, the data obtained in the fieldwork campaign between 30 April and 22 June were presented, a period included in the State of Alarm decreed by the Spanish government, for which the confinement was established, which would begin on March 14 of that same year [23].

The validity of the construct was calculated, defined by Kaiser–Meyer–Olkin Measure (KMO) of Sampling Adequacy, that must be >0.7, and the Bartlett’s Test of Sphericity, whose value must be ≤0.05. These results are shown in Table 1.

The KMO is 0.921, thus it is considered correct. The Bp = 0.000, so it is also correct.

### 3.1. Quantitative Results: Questionnaire

**Figure 1:**
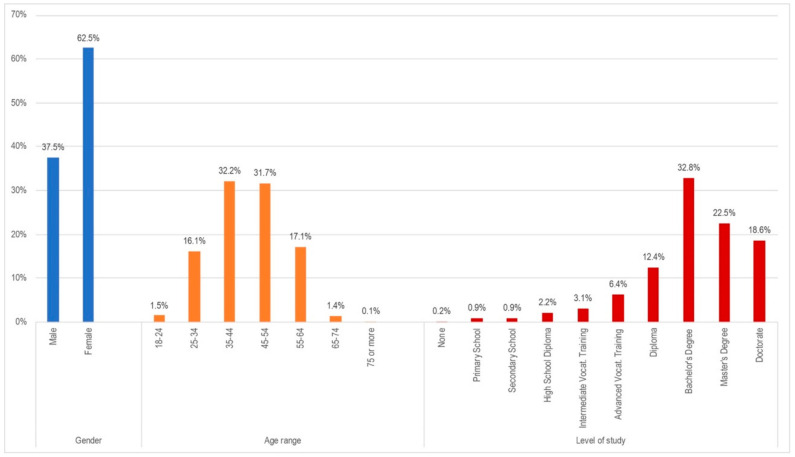
Sociodemographic data of the teleworker-households sample.

During the data collection campaign by the participants, 1271 responded to the telework question. Of all of them, 1170 declared to be teleworker households. Of this sample, 1054 participants answered as teleworkers in the first person. Figure 1 reflects the socio-demographic distribution from the obtained home-teleworking sample.

The sample mainly presented middle-aged individuals (35–54 years), mostly with university experience, and with a greater representation of women.

**Figure 2:**
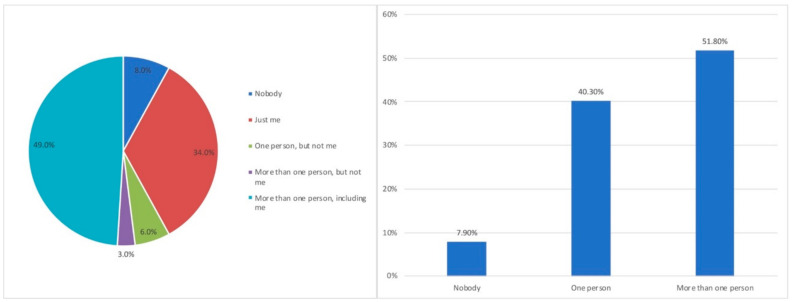
Distribution of people teleworking/tele-studying in homes, as requested in the questionnaire (**left**) and grouped by number of people (**right**).

Figure 2 shows the distribution of homes in which teleworking or tele-study was carried out during confinement.

From the observation of Figure 2, it can be concluded that 92.1% of the participating households had at least one member carrying out telework or tele-study tasks. Furthermore, 83% of the participants who answered this questionnaire did so in the first person, as they had been teleworkers in their homes during this period of isolation. Likewise, in more than half of the households surveyed, more than one person teleworked or tele-studied, which is a relevant fact.

#### 3.1.1. Descriptive of Variables by Frequencies

**Figure 3:**
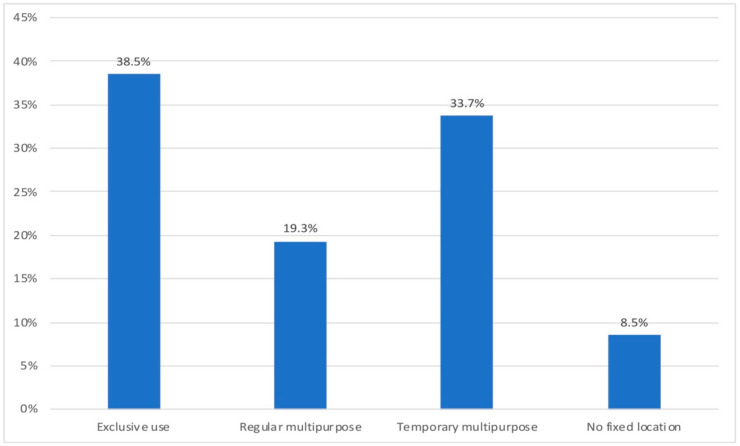
Availability of a usual space for teleworking.

Of the 58 questions in the questionnaire, five were directly related to teleworking. The first of them is the one reflected in Figure 3. Other sociodemographic and housing data and the way of living in confinement completed the original questionnaire. Figure 3 shows the results by frequencies on the availability at home of a usual space for teleworking.

The results from Figure 3 show that 42.2% of homes did not include any kind of workspace before lockdown. Those teleworkers had to make room for this task during the lockdown (33.7%), or roaming around the home, according to the household’ circumstances or spatial demands. 57.8% did have a previous room, either exclusive (38.5%), or shared with other cohabitants and/or uses (19.3%).

**Figure 4:**
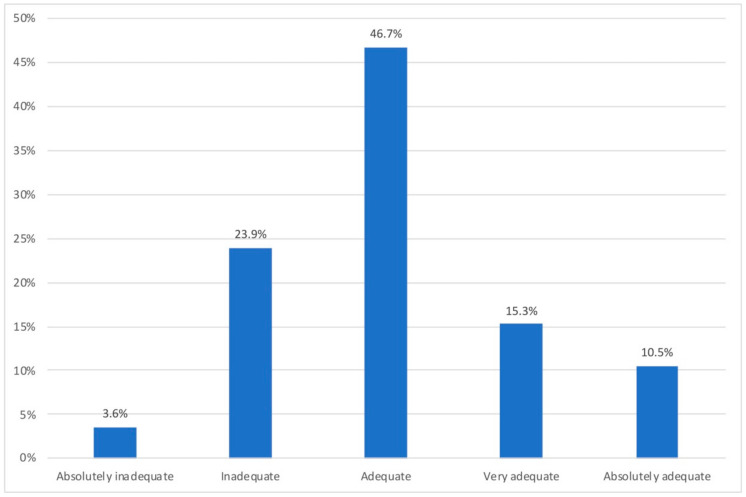
Adequacy of the telework space.

Figure 4 shows how the space in which the participating households teleworked was considered.

As shown in Figure 4, 72.5% of the sample was considered at least adequate and 25.8% being very or absolutely adequate. Slightly more than a quarter of the sample considered their home workspaces inadequate.

**Figure 5:**
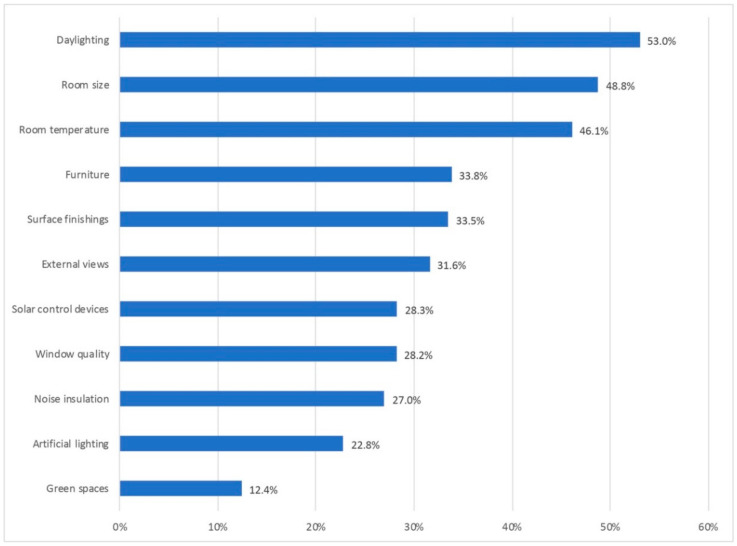
Aspects valued as good/adequate of the workspace according to the participants.

Figure 5 shows the aspects valued as good or adequate of the workspaces in the participating homes. This was a multi-choice question.

According to Figure 5, the aspects that participants considered adequate the most in their telework spaces were daylighting (53%), room size (48.8%), and room temperature (46.1%), followed by furniture (33.8%), surface finishes (33.5%), and external views (31.6%). After that, the general quality of windows, including their solar control devices (28%), and noise insulation (27%) were also valuated as suitable.

**Figure 6:**
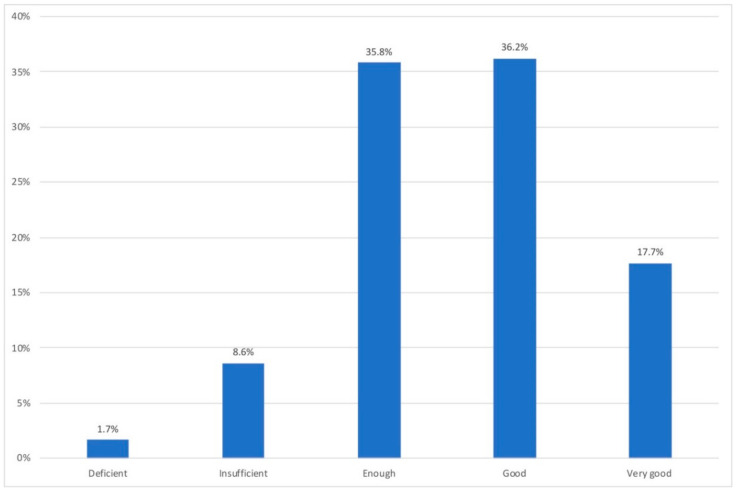
Qualification of digital media (resources, devices, etc.) for teleworking.

Another aspect that was assessed through the questionnaire was the digital media (resources, devices, etc.) available to telework from home, as shown in Figure 6. In general, people were satisfied with them, with 53.9% considering them good or very good. Just 10.3% of the sample declared not having appropriate digital resources in the home to develop telework, and 35.8% valuated this equipment just “enough” to that aim.

#### 3.1.2. Characteristics of Households and Dwellings According to the Assessment of the Teleworking-Space Adequacy

Next, Table 2 describes the characteristics of cohabitants’ nucleus and houses and their relationship with the appreciation on the telework-space suitability.

In this table, the adequacy of telework spaces was crossed with household and dwelling characteristics. The sample of teleworkers who gave their opinion on telework space adequacy, was mainly middle-aged persons (63.7%), with university studies (87.1%). The sample habitat was urban for 75.5% of cases. On the households’ composition, half of them had 3 or more members, while 22.2% lived alone, and 51.6% lived with minors under the age of five.

On the dwelling characteristics, 71.5% of households owned their houses, 77.8% being multi-family types. The house size was shared among stablished ranges, highlighting 38.5% of 61–90 m^2^, a quarter of 92–120 m^2^, and a fifth over 120 m^2^. The overall lighting of homes was adequate for 60.3%. The air quality was good/very good for 87.2%. The home was not or little noise insulated for 39.1%.

Related to the adequacy of telework spaces, this perception was better for older people, living alone, or at least with no children under 5, and higher completed studies, and urban habitats. Those workspaces were better valued in owned and single-family homes, increasing their suitability positively with the floor area. Workspace features such as overall lighting, air quality, and noise insulation helped to the better appreciation of the whole working area.

The results of Table 2 also show those characteristics of the households and dwellings that were related significantly with the general perception of teleworking-space adequacy of the participating homes.

According to the home characteristics, age and coexistence with children under 5 affected the adequacy of the telework space, being more appropriate at an older age, and in case of not living with these minors.

Regarding the house features, owning regime of dwellings, with more useful areas, especially if they were single-family, were statistically related to adequate teleworking spaces.

In order to delve into the relationship between the cited variables such as age, home ownership, completed studies, and living with children under 5 years of age, an analysis called the Classification Tree was carried out. For this analysis, the software SPSS, version 26, was used.

This test looks for causal relationships around a dependent variable and shows how strong these relationships are. In addition, it searches through algorithms for relationships with the variables provided, in such a way that it offers results in branches. If the relationship supports more variables, this branch will show it, searching for relationships until it finds others. As a result of this search, it was established that the determining variable for the causal relationships of the adequacy of telework spaces was the housing tenure regime. After it, the strongest relationship is established for homes owned, and within these, for those people over 55 years of age. However, it did not rule out those home-owning households with younger age members, but without the presence of children under 5 years of age. Finally, in the case of being young owners and with children, the causal relationship, although weaker, would occur for those with completed university studies.

Following the variables described in Table 2 and related to the internal environmental quality of the home, a well-lighted home, with better air quality, as well as good insulation against noise, were associated with more adequate teleworking spaces.

Those variables that did not show statistically significant relationships with the adequacy of the teleworking space in the home were: educational level, number of people in the home, and habitat.

To validate the results in Table 2, an adjusted binary-logistic regression was carried out, taking into account the dependent variable “Adequacy of the Telework Space”.

The overall percentage correctly classified: for Table 2, the percentage was 63.1%, thus >50% of cases were correctly classified, and the model was accepted.

For this logistic regression, all the variables shown significant relations, and all but one operated positively (the exception was “persons in the home”). The strongest relationships between independent variables and the dependent one, were given for “dwelling air quality” and “overall lighting of housing”, followed by “housing type” “persons in the home”, and “dwelling useful floor area”.

#### 3.1.3. Aspects of the Teleworking Space, According to Their Perception of Adequacy

The aspects related to telework spaces according to their conditions, resources, and physical features are listed and their relationships were tested statistically, as shown in Table 3.

Table 3 lists aspects of teleworking as an activity, and specific characteristics of the teleworking space, with the perception of such space as “adequate” by its users.

The sample of teleworkers in their homes, shared this activity with other household members for 58.5% of cases. The workspace was exclusive for 38.4%, multipurpose for 53.1%, and no fixed for 11.8%. Digital resources were good/very good for more than a half, enough for more than a third, and insufficient for a tenth of the sample.

The room size was considered suitable for 72.8%, and its temperature for 68.8%. Daylight was valued positively for 78.9%. The window quality was not adequate for 57.9%, while the noise insulation was considered inadequate for 59.9%. Artificial lighting was considered not good in 66.1% of cases, and the views to the outside were not good for 53%. The furniture was good for 50.4%.

Related to the adequacy of telework spaces, this perception was good for people teleworking exclusively in their homes, having exclusive workspaces or regular multi-use environments, and with proper digital media. Telework spaces were better valued if each of their characteristics (room size and temperature, daylight entrance, window quality, noise insulation, artificial lighting, furniture, and outside views) were suitable.

One of the included variables did not have a statistically significant relationship with this dependent variable. It was the number of people who teleworked or tele-studied at home.

Regarding the space for teleworking, the most exclusive spaces and those established in a fixed (pre-pandemic) way were perceived as the most appropriate. Similarly, homes in which digital resources were better defined the telework spaces more positively.

On the other hand, those larger spaces, with comfortable temperatures, with natural light, and with quality windows (including solar control elements), were perceived as more suitable. Also, those better insulated against noise, with good artificial lighting, better views to the outside, furniture more in line with the task, better surface finishes, or spaces for vegetation, were also perceived as adequate.

Again, to validate the results in Table 3, an adjusted binary-logistic regression was carried out, taking into account the dependent variable “Adequacy of the Telework Space”.

The overall percentage correctly classified: for Table 3, the percentage was 77.9%, thus >50% of cases were correctly classified, and the model was accepted.

For this logistic regression, all the variables shown significant relations, and operated positively. The strongest relationships between independent variables and the dependent one, were given for “space for teleworking” and “daylight entrance”, followed by “room size” “furniture”, “digital resources”, and the remaining ones.

#### 3.1.4. Telework Space Adequacy Index (TSAI)

Table 4 shows the Telework Space Adequacy Index (TSAI). This index relates different aspects of households and dwellings with characteristics of actual telework spaces that participants perceived as adequate in their homes. This index was constructed as a ranking (from 1 to 10). Starting from the multi-response variable, exposed as follows: “Please indicate which of the following aspects of your workspace seem good or adequate (if you do not indicate them, it means that they are insufficient or inappropriate. You can indicate more than one answer)”, 11 potential aspects of the actual teleworking space were offered.

The construction of this index is based on the following principle: “The more aspects of the telework space considered good or adequate, the better the overall assessment that can be made of that space”.

The 11 potential aspects were grouped, to later separate them in “essential” and “not essential”. The essential ones were the five related to aspects of building design and construction quality (room size, room temperature, natural light, noise insulation, and quality of windows). The non-essential ones were referred to those characteristics that are more easily modifiable by cohabitants (artificial light, solar control devices, surface finishes, furniture, outside views, space for vegetation). Thus, Table 4 shows which general variables in household composition and dwelling features were statistically related to perceive a workspace as more or less adequate, attending to their mentioned aspects. For an easier way to understand this index, it was expressed as a ranking, to resemble it to a score. Higher ones (>5) indicated that the related independent variable collected more adequate workspace aspects, so that the general workspace valuation was considered better. Otherwise, when a score was under 5, the valuation of the general workspace was considered worse, based on fewer aspects valued favorably individually.

The independent-samples Kruskal–Wallis test was used to calculate these relationships. The significance level was stablished for *p* < 0.05.

The colors assigned to each cell are related to the score range, to easily detect which variables were linked to fewer or more aspects considered adequate in the real workspace at home, and thus, also associated with a more adequate telework space in general.

Regarding the Telework Space Adequacy Index (TSAI), it was broadly observed that, of all the statistically significant variables crossed with this grouped telework space aspects (education level, housing tenure regime, useful surface area, adequacy of space telework, type of home, and usual work activity), there were significant differences in their scores according to the different response options offered, so, depending on the home characteristics, the space could be perceived as adequate (≥5), or inadequate (<5). The grouping of non-essential aspects achieved fewer points in the ranking compared to the grouping of essential features of the telework space. It indicated that the presence of the latter helped a more positive assessment compared to the so-called non-essentials, being more relevant aspects. That is, daylighting, room size and temperature, noise insulation, and window quality were all effectively considered essential characteristics and contributed to a higher index value. The other features were not as decisive when evaluating a telework space (in fact, none of their evaluations exceeded the value of 5, which would be the minimum adequacy score out of 10). Relationships were established as more positive for higher levels of education, also associating with public servants and self-employed jobs. Regarding homes, those owned, with a larger surface area, preferably single-family, were better perceived. The adequacy of the telework space in general demonstrated its statistical relationship with the telework aspects grouped and perceived as adequate, although it was established more clearly for the most essential aspects, as in the rest of the relationships between variables.

The lowest value of the index was obtained for unsuitable teleworking space, or not very suitable despite meeting non-essential characteristics, while the highest value of the index was reached for dwellings with larger surface area (>120 m^2^), in whose teleworking spaces meet the essential categories.

To test the index reliability, the Cronbach’s alpha was calculated, obtaining the general value 0.887 (>0.7). When any item was deleted, no single value significantly altered the general value. Thus, it can be considered as reliable.

### 3.2. Qualitative Results: Photographs

**Figure 7:**
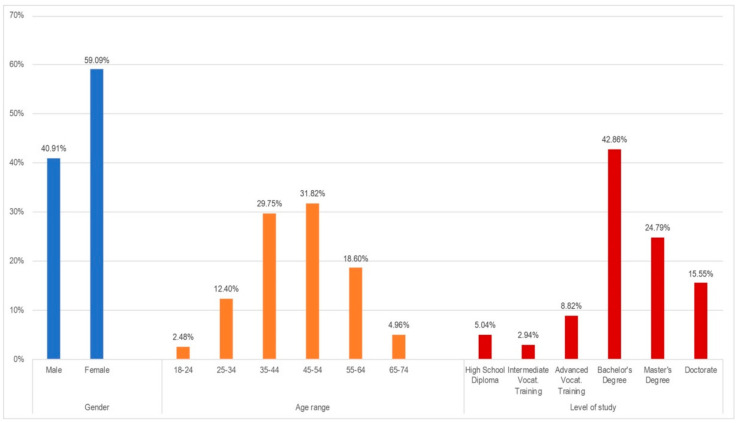
Distribution of qualitative participation by gender, age range, and qualification.

For the qualitative approach, based on photographs and written testimonies, 785 responses were collected. Figure 7 shows the distribution of households who participated in the qualitative part, by gender, age range, and qualification.

**Figure 8:**
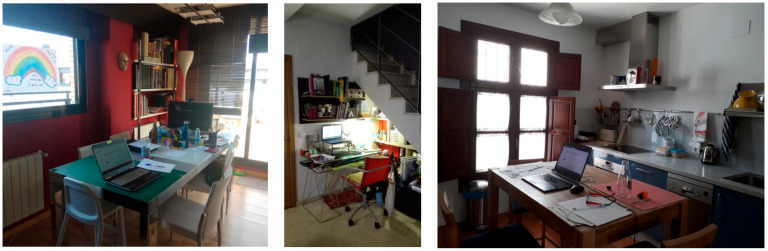
Photos taken by participants on telework spaces.

Among all the responses to the qualitative questionnaire, 605 images were obtained, of which 213 belonged to telework spaces. Some of them are shown in Figure 8.

Categories and subcategories extracted from the graphical information are in Table 5.

From the classification carried out through the graphical analysis of the photographs, and their subsequent categorization, interesting questions were extracted (Table 5). They might give an answer to the results obtained in the quantitative part, in terms of the perception of the telework space adequacy, the distribution of adaptation of the different characteristics of these spaces, and their connection with the circumstances of the home and the characteristics of the dwellings. These characteristics were analyzed based on the level of detail intended by the author, and therefore on the information shown in each one of them.

In the first place, it is shown that the places most used for teleworking were, equally, either the living room and the studies/offices, followed by the bedrooms and living rooms. Therefore, they have not emphasized in a forceful way the exclusive spaces, but rather spaces shared either with cohabitants, or with other uses, before or during the confinement. This is related to the classification according to the use of teleworking space, where shared spaces (fixed, and later circumstantial) stood out, followed by itinerant teleworking, and lastly, those for exclusive use.

As for other characteristics of the room in general, the larger spaces highlighted (which could be related to the room chosen to telework), although not far from the perception of small rooms (92 vs. 83). The lighting perceived in photographs was mostly natural (145) and adequate (143), although in many cases lamps (48) and/or inadequate lighting (41) were also seen. This could also be nuanced by the time the photo was taken (it could be at night), or the use of solar control elements, which was seen in numerous photographs (104). In relatively few photographs, exterior views were appreciated, highlighting views of other buildings (27), nature (20), or own exterior spaces, such as patios (16).

As a joint assessment of the telework space from the observation of the photos, those not or little adequate stood out (80). After these, a total of 57 were presented as very or totally adequate, and 50 were assessed as adequate. For this purpose, the furniture used, the availability of a clear work area, and digital resources were observed. Subsequently, these aspects were evaluated individually.

In general, digital resources were good or very good, or at least sufficient, in the same proportion (84–83), while few households reported deficiency of these means (16).

The area available to work turned out to be small for most of the spaces (105), compared to the amplitude shown in 79 of them. Regarding the spatial organization, the vast majority were tidy (120 vs. 59), although highly loaded spaces (108) stood out compared to those clearer (75). This may be related to the availability of auxiliary furniture, which stood out as insufficient (78), although in 99 households there were at least enough or even adequate ones.

Regarding ergonomics, this in turn was subdivided according to the suitability of the furniture (chairs and tables), the type of digital resource available, and auxiliary elements that would improve this aspect. Specifically, chairs were in the same proportion adequate (73) and inadequate (72). The tables were, for the most part, adequate (108 vs. 69), although it should be noted that the adequacy of the tables was perceived as including a minimum in terms of suitability. Perhaps a possible reason for highlighting the amount of tables compared to the chairs is the cost of acquiring one or the other, although this is only an assumption. Finally, regarding digital resources, the majority had laptops, not computers (117 vs. 70), although these resources were partially improved with auxiliary elements. Laptop is not an ergonomic measure, although it presents important benefits of another nature, possibly also the easiest resource to obtain on the part of the employer or of own acquisition of homes. Also, it allowed for mobility in the house, which would ease flexibility to conciliate and care for other cohabitants, the alternation of uses with the household members, or the adaptation to spaces with other characteristics more suitable for teleworking, like sunlight, good views, or comfort, for example.

In 31 cases, it was evidenced that teleworking spaces were being shared with more members of the household, regardless of whether they were minors or not.

Regarding other elements perceived in the photographs, decor stood out (112) as a factor that could make the perception of the space more pleasant (as it is personalized and therefore more pleasant), followed by the presence of heating elements (41), plants (32), and children’s objects in 28 of the cases analyzed. Carpets and organizer panels were also observed in a minority.

### 3.3. Qualitative Insights: Storytelling

Regarding the narrative discourse, the labels related to the photographs can be distinguished (3 labels for each one of the images), of which 595 were obtained for teleworking. For its classification and treatment, the NVivo software was used, creating the word clouds from the analysis of frequencies by terms.

Table 6 presents the word cloud corresponding to the image tags for the telework issue of the photographs, reflecting the ten most found terms, and their frequencies.

The word frequencies indicated that physical environmental aspects such as the lighting (specially daylighting), the amplitude of the working area, the furniture, digital resources, or comfort were highlighted. Living rooms also stood out. This information supports the results obtained in the categorization of photos, given in Table 5.

In addition to the tags, the photographs were accompanied by the answers to the five questions about the image, its context and its intention when making it.

In relation to the questions that accompanied the photographs, the last two especially asked about the status quo of the current context of the teleworking space, and on the final reflection (how people’s lives could be improved), through the message (lessons learned).

As regards the whole qualitative analysis, the most recurrent terms were related to the activity itself, the location, the lighting, the availability of space, the digital resources, the furniture, and the qualities of this space, such as comfort or size. Graphically, very different workspaces could be observed in the images. Most notably, those spaces occupied on an occasional basis, in most cases shared by other people in the home, such as living rooms and dining rooms, and to a lesser extent, bedrooms and even kitchens. These spaces often share certain characteristics: they are spacious, with good natural lighting and ventilation, sometimes with pleasant views, which supports what was obtained in the questionnaire about the majority adequacy of teleworking space. However, these characteristics have been achieved by sacrificing aspects such as ergonomics (using laptops, or dining room furniture), privacy, or isolation from other cohabitants, for instance.

### 3.4. Comprehensive Analysis: The Mixed Approach

Bearing in mind all the managed data sources, it highlighted the coherence among the findings. Nevertheless, the information obtained from the qualitative approach, is mainly useful to understand the quantitative results, with a clear explanatory function.

In the first place, all the types of scenarios declared through the answers to the close-ended questions were observed in the photographs, in terms of teleworking spaces. Analyzing the photos in more detail, with the help of their categorization, in contrast to the quantitative questionnaire, the high percentage of suitable telework spaces declared quantitatively stands out. Also, the main characteristics attributed as adequate to these spaces, i.e., natural light, room size, temperature, furniture, and finishes or views, or solar control devices, stand as the most prominent.

At significant crossings, well illuminated spaces with good air quality and noise insulation are effectively valued. However, in the photos it can be seen that the most used spaces were living rooms and studios or offices. So, what was going on with these valuations? How can exclusive spaces for teleworking be equally valued as antagonistic, shared, more social, and unspecialized spaces for teleworking activity? With all the information obtained, it could be established that the members of teleworking households sought, according to their different circumstances both in the family and in the possibilities of housing, solutions that were as balanced and appropriate as possible. Prioritizing aspects such as lighting, the amplitude of spaces, ventilation, or isolation (whether it was to interior noises as well as exteriors to the house), they resorted, in the absence of specific telework spaces, to the amplitude of the rooms.

On the other hand, in many cases they had to combine childcare with work, which is why these large spaces were suitable for grouping the household. But in the assessments of adequacy, they were worse perceived. In the photographs, most of the spaces shared with other uses appeared, both those created before the pandemic and those that arose with confinement, which were followed by itinerant spaces, and lastly, exclusive fixed ones. In these large spaces, in addition to natural light, there was also the ability to tone it down and control it with solar control devices, which was very interesting to avoid the unwanted effects of lighting excess, such as glare.

Despite the spaciousness of the rooms, the effective work area was very small. The spaces were tidy, although heavily loaded with documents and elements related to telework. Those spaces with poor or non-existent auxiliary furniture were highlighted, to expand the effective telework space.

Furniture was the fourth characteristic voted as adequate in the telework spaces of the participants. However, evaluating the photos, these spaces were mostly unsuitable in this regard, as they suffered from inappropriate chairs and/or tables, in a greater proportion than desirable. The tables were mostly adequate, although there were a large number of invalid ones. The chairs were equally adequate and inadequate. This may also show a possible error about the perception of the furniture suitability by the participants, from an ergonomic point of view. In this sense, there was an excess of use of dining room furniture to exercise telework, or, in general, non-specialized house furniture. Also in this sense, the use of laptops, non-ergonomic devices, for continuous use over time and for many hours a day, were highlighted. These digital resources were appreciated as sufficient and good to a great extent, but more from a functional than postural-hygiene point of view.

On the other hand, this adaptation of the telework space has shown in a forceful and validated way to be linked to a series of very specific socioeconomic characteristics, which could be linked to a certain status of people, in a vital moment of greater stability. Its adaptation to a mature age relates to the fact of not living with small children, to having a spacious home, owned, possibly single-family, and with good environmental qualities (natural light, air quality, and noise insulation). These better perceived telework spaces corresponded to exclusive spaces for this purpose, and with better individual characteristics. These were: natural light, size, temperature, artificial lighting, windows, insulation, furniture, and views. The worst perceived aspects were related to being young adults, the presence of young children, living in rented houses, and smaller ones. These teleworkers often had to adapt shared spaces, with the use of inappropriate furniture being perceived in the photographs. Sometimes the presence of children’s objects was noticed, or evidence of very small effective work areas also shared with other members of the household, and even rotating their own digital devices with others. The perception of these spaces was obviously much worse.

These evaluations are also supported by the contents of the word clouds, or those that were most used in the written testimonials. Again, the prominence of natural light, spacious, comfortable spaces, as well as the provision of resources such as furniture in relation to the telework space were highlighted by the participants.

All the cited household and dwelling characteristics were statistically related to the adequacy of the telework space both through bivariate relationships and through the Telework Space Adequacy Index (TSAI). In the latter, the best valuations were stablished for stable labor positions, such as civil servants and entrepreneurs, in owned, larger and single-family homes, again. The essential characteristics of these spaces, according to teleworkers, were those that most affected these perceptions of adequacy, such as natural light, room size and temperature, noise insulation, and the quality of windows. Interestingly, these essential characteristics, of all those facilitated, depended on the design and construction of the building, and that could only be modified, in addition, by homeowners, not by tenants.

Through these analyses, the individual aspects of the telework space were significantly related to the global perception of the adequacy of these spaces, in a positive way (the more appropriate aspects, the greater the overall adequacy of the space). The so-called essential aspects were distinguished, and their greater entailment is observed with the best evaluations of the teleworking space adequacy, especially, with dwellings larger than 120 m^2^.

Therefore, the analysis was presented as coherent, and the nuances provided by each of the approaches allowed a better understanding of the results, enriching the global discourse on what teleworkers understood and needed as appropriate in their telework spaces, both as a global assessment, as in its different aspects.

## 4. Discussion

The possibility of teleworking is determined by the type of work, the company capacity to facilitate this work development remotely, in addition to the home characteristics, technical means, and the capacity of the job to be carried out in that way [15].

The study presented seeks to offer a holistic and complete vision of the degree of adequacy of homes to telework, and more specifically in the context of confinement due to the COVID-19 pandemic, decreed for Spanish households during the spring of 2020.

To our knowledge, there are no prospective or exploratory studies to date that assess the adequacy of the telework space from the housing point of view, assessing in detail those aspects, both sociodemographic and on dwelling characteristics, which gave an idea of what affects having an adequate workspace at home. The approach through the mixed method allowed us to not only be able to collect information through surveys, but also to have graphic and written testimonies about the perception of teleworkers of their own telework spaces and what characteristics they understood as adequate to have a work environment comfortable at home.

According to a study carried out in 11 countries, the employees who prefer to telecommute on a daily basis are women (64%), the main reasons being family reconciliation for family care (61%) [43]. The present study found a predisposition in women to telework. However, not all the home circumstances could be taken into account, due to the extension of the study and the quantity of data offered. For instance, a deeper study of female teleworkers with children, for instance, and their perception of telework and workspace, were not further analyzed. It could be considered for future investigations, since many official resources and documents reinforce the importance of the role of gender, the pandemic, and the influence of telework.

The images have in general great narrative potential per se, containing a lot of information in themselves that would otherwise be almost impossible to count in a reasonable space. This also allows people with difficulties to express themselves, for example non-literate persons, to explain their reality mainly based in photos, with little support of oral testimonies, but with hardly any culture [44].

Another advantage it has is the specific objectification of the subjective, graphically showing numerous sensory effects (for example, light reflections that in turn cause a certain effect, desirable or not), more or less annoying, that they remain in the snapshot, and thus a third party can evaluate them for himself [39]. However, the intention of the author cannot be ignored when taking the photograph itself. This is why the participants were deliberately asked to take the photograph, tag it with keywords, and answer the questionnaire of five open questions, which led to reflection on what to photograph, what to show, why they did it, and what could learn others from them and the situation reflected [45].

The sample obtained from teleworkers at home was very high in this study, and it stood out that in more than half of households there were two or more people teleworking. This was valued positively for the purpose of the analysis itself, which was to know first-hand what their experience had been in relation to the space and available resources, and their quality. Although it must be taken into account that this was due in part to the very method used to disseminate the survey, online, which was very recurrent in these studies contextualized in confinement. This fact marked a bias in the participating sample, since certain minimum digital resources and the internet were necessary to access and fill it, the same ones that were needed to approach work from home, in addition to the selection bias due to the way of disclosing the study, which could not be randomized due to the lack of resources. This fact could affect some sample characteristics, such as the distribution of working activity, or level of completed studies. Also, the certain household composition and some age ranges could be somehow overrepresented. However, this has been an opportunity to address the taxonomy of the telework space and its characteristics, having obtained such a high sample for the national territory, at least in the context of confinement.

The quality of these spaces was statistically significantly related to having a higher quality home, in general terms, more spacious, single-family, owned, and being 55 years of age or older (an age at which it is more likely to have socioeconomic and labor stability, in active period, which leads to greater satisfaction or well-being [46]).

Of the entire sample of teleworkers available, 42.2% had to find a place to telework. However, only a quarter of all of them found this space little or not suitable. The aspects that have been most valued of the teleworking space available were the daylighting, room size and temperature, furniture, surface finishes, and the views to the outside, mainly.

On the contrary, those who found the space more inappropriate were, to a greater extent, young people, living with children under 5 years of age, and mainly in rented and smaller houses, flats, without a fixed place to telework, and deficient digital resources; with poor indoor environmental quality (lighting, air quality, and noise insulation), and whose spatial qualities were not adequate either.

This was in line with what was observed in the photographs, where mostly natural and adequate lighting predominated, as well as mostly wide spaces, although they often presented signs of the presence of children, and whose furniture was far from being ergonomic and adjusted to the needs to work from home continuously. On the other hand, the photographs showed very diverse digital resources, being mostly laptops, which also did not favor ergonomics and postural hygiene, in favor of possible roaming, either in search of better environmental conditions, or due to the need to take care of children, taking into account that in this period the schools were closed [47,48].

This would also be explained by the fact of a strong greater response from women, since the studies carried out not only at the national level, but also at the European level [49] and international [50] on gender and work at home, especially in the context of COVID-19, indicated the predisposition of women to stay at home and to care for dependents [51,52,53], to telework, or to reconcile both circumstances [54,55], which would have been aggravated by the public health measures related to home confinements [56,57,58]. Other majority characteristics of the study sample, such as the level of university studies, the average age (active), and the high rate of spaces suitable for teleworking, support the idea that teleworking is a work modality that can be mainly afforded privileged people [14]. Although the COVID pandemic has further diversified the profile of teleworkers, expanding them to other less qualified workers [38], these arguments are equally valid and continue to be the majority, apparently more among sectors where there is a greater presence of the female gender [59].

The scarcity of studies that analyzed telework in general is striking, and even more so the taxonomy of its available spaces, especially in confinement, given the circumstances of the presence of the entire cohabitants’ nucleus in the home. While teleworking in general is a mostly temporary or sporadic practice or sometimes based on specific circumstances, in any case it has been recently adopted not only at the Spanish level, but also at the European level [60], which, however, was drastically assumed during confinement, and which seems to have signs of an upward trend [60,61], which has even led to the revision of the regulations to include it as a labor modality in Spanish legislation [30].

Among the advantages of teleworking in people’s lives, those who defend its benefits for reconciling family and work life, and even personal life, stand out [62], and although there are studies that report benefits in terms of performance and productivity [63], especially during confinement [64], there are also those who weigh other aspects that could lead to another series of deficiencies or problems, be it lack of concentration, time organization, conflicts within the home due to the family-work conciliation itself [22], greater dedication and difficulty to disconnect [65], isolation, or lack of the benefits of social contact in general, including leadership tasks [66], or face-to-face contact with colleagues in particular [67]. In this sense, precisely because of caring for minors and work-family reconciliation [68], some female teleworkers reported worse productivity compared to their male colleagues [69].

Other advantages have been theoretically attributed to the relocation of work [70], and therefore to the elimination of unnecessary motorized movements [71], in favor of other modalities such as walking [72], cycling [73] or running [74], which in turn would lead to an environmental improvement by reducing the consumption of fossil fuels, and emissions associated with mobility and the pollution it generates [75,76]. In turn, the decrease in travel would lead to an improvement in the quality of life, derived from the saving and redistribution of time, dedicating it to family or leisure [77]. Decentralized city schemes through models such as Barcelona’s superblocks [78], or population redistribution in peripheries and less dense cities could also in turn have economic and environmental impacts on pressure in large cities [79], and at the same time socio-sanitary, since the offer would be broader and cheaper in general, favoring the recovery of these marginal urban areas, and other intermediate and even rural territories [80], which for this, should be appropriately endowed at both an individual level (by supply networks, including the internet, stable and with adequate supply), as well as social (infrastructure, social, health and public interest services, etc.) [81].

In the last year certain studies have questioned some of these supposed benefits [82]. Be that as it may, for obvious reasons, most of these improvements did not arrive in time during the confinement, since this was not expected or imaginable on a global level, so teleworking in this context can be understood as a kind of “test” or experiment, in unusual circumstances. That is why it opens now, after the “new normal” [25], an opportunity for the organization of all interested parties to adopt the necessary measures in terms of legislation, worker protection, company conditions, and adaptation of households to telework, thus ensuring the minimum conditions of safety and health, including ergonomics, without undermining other aspects such as protection and guarantees towards the worker, their rights, such as their duties and conciliation with family life [83]. This would also affect the post-pandemic housing discourse, which should include teleworking among its potential activities, which could carry other connotations, normative, spatial, distributive and functional, in favor of resilience and adaptation to new times [5].

## 5. Conclusions

The main conclusion that can be drawn from this article is, on the one hand, the uniqueness and exceptionality of teleworking in homes as a result of the confinement due to COVID-19, which caught at least 42.2% of teleworking households off guard of the sample, who did not have a fixed site to carry out this task. On the other hand, observing the characteristics of the sample, those who did have fixed telework spaces had a certain sociocultural status and stability, compared to those who did not have these spaces, who used to respond to a profile of a young adult, with young children, for rent, living in smaller flats.

Likewise, it was related to a good telework space: having a better, more spacious home, owned, with a certain age (over 55), and having good indoor environmental quality in general (air quality, lighting, and noise insulation).

After the results were triangulated [84] and validated with different tests (The Classification Tree and the Binary Logistic Regressions), the adequacy of telework spaces was significantly related to those household and dwelling characteristics, with a strong causal relationship among them. Therefore, it could be said that having an adequate space for teleworking in the home is associated with a certain privilege, social status, and socioeconomic stability (also associated with a certain age and family circumstances, that is, to own a house, be over 55 years of age and therefore with less probability of presence of minors, and households with fewer people), reserved for certain social classes. On the other hand, the characteristics of telework itself and of the spaces intended for this purpose were: having adequate digital resources, having exclusive spaces to undertake this task, and the so-called “essential” aspects, that were, those that depended on the design of the homes, or the state of conservation, and where appropriate they could only be improved with a retrofitting intervention [85], not depending on the cohabitants, as occurred with the “non-essentials”, both classifications contained in the telework space adequacy index (TSAI).

Teleworking has been one of the most disruptive activities during confinement in Spain. According to official sources, it has been partially or totally carried out during the pandemic for up to 57%, with 23% attending the office, while a fifth have worked from other locations [49]. One possible reason for the apparent overrepresentation in this study has been the bias inherent in accessing the study forms through the internet [86]. It should also be considered that this study has included all the individuals who tele-studied at home, including minors. In the original questionnaire about dedication to different activities, the perception of temporary dedication to telework and study stood out above the rest, which confirm the above. It should be noted that more than half of these households claimed to have more than one person in this situation.

Obviously, these circumstances should be reviewable for a normal teleworking context, since the presence of minors or not, under normal circumstances should not be a differentiating feature, to name a few. Despite the observation of these types of characteristics, very remarkable judging by the results obtained from both the quantitative and especially the qualitative approach, much more impressive and loaded with symbolic information, it is relevant to document what the current state of the housing was in Spain regarding this booming work modality, and what its main defining housing and environmental characteristics were, the aspects most valued by teleworkers, and the potential areas for improvement.

According to the aspects better valued for the telework spaces, and the socio-economic characteristics of teleworker households, this analysis has greater implications in housing design, when telework, as new domestic activity must be collected in the architecture residential discourse. Within a “new normal” or post-pandemic paradigm, where more flexible, resilient and open to different adaptations of home-schemes must be included, the possibility to remote work or study has to be enough room, not only physical, but figurative. In addition, in the renovation wave emerging in recent years, and driven by Europe, more effective plans and strategies must be made to “leave no one behind”. Some of the greater concerns related to habitability, comfort, energy dependence [87], noise insulation, and indoor air quality, have direct influence not only in people’s well-being and health, but also in the perception of what is expectable for a good workspace [88]. Thus, in the emerging debate of how to ensure a good work environment in the homes, guidelines and good practices to get an adequate workspace have to be given, and when necessary, providing all the resources to employees, to guarantee a safe and comfortable working time. Yet, it must be in balance with other aspects that affect the personal and private space, also depending on the household and home features.

In this sense, to favor the implementation of teleworking in the home, there must be not only a consensus on how to adapt the measures at home. The responsibilities and rights of each party (employee and employer) must also be made clearer. Moreover, depending on the affected sector (companies or employers), governments, public administrations, and other social collectives must support them, providing resources or even promoting alternative measures to ensure teleworking for families, as well as family and work conciliation in the best possible conditions, especially, but not exclusively, in extreme situations like COVID-19 confinement.

From a housing point of view, both designing new residential buildings and when retrofitting the existent ones, this new activity must be implemented as a domestic potential task, and therefore to include it in its functional program. Also, to carry out this activity properly, building codes have to be created and/or modified in order to improve the housing envelope [89], infrastructure, and quality. Also, efficient design/renovation housing strategies to incentive this functional inclusion must be adopted and applied.

This study hopes to contribute to the task of generating knowledge and promoting the guarantee of future compliance with these aspects, as far as possible.

## Figures and Tables

**Table 1:** Validity: KMO and Bartlett’s Test.

KMO and Bartlett’s Test		
Kaiser-Meyer-Olkin Measure (KMO) of Sampling Adequacy		0.921
Bartlett’s Test of Sphericity	Approx. Chi-Square	7659.537
	df	55
	Sig.	0.000

**Table 2:** Characteristics of households and dwellings and the adequacy of the teleworking space.

Variable	Total N (% col)	No or Little Suitable N (% Row)	Suitable/Very/Totally Suitable N (% Row)	*p* *
General	1121 (100)	308 (27.5)	813 (72.5)	
Age range				0.006
18–34	192 (17.2)	63 (32.8)	129 (67.2)	
35–54	710 (63.7)	203 (28.6)	507 (71.4)	
≥55	213 (19.1)	41 (19.2)	172 (80.8)	
Education level				0.157
Undergraduate	144 (12.9)	45 (31.3)	99 (68.8)	
Graduate	510 (45.6)	149 (29.2)	361 (70.8)	
Postgraduate	464 (41.5)	114 (24.6)	350 (75.4)	
Habitat				0.159
Urban	838 (75.5)	222 (26.5)	616 (73.5)	
Not urban	272 (24.5)	84 (30.9)	188 (69.1)	
Persons in the home				0.398
1 person	240 (22.2)	58 (24.2)	182 (75.8)	
2 persons	287 (26.6)	80 (27.9)	207 (72.1)	
≥3 persons	554 (51.2)	160 (28.9)	394 (71.1)	
Live with minors (<5 y.o.)				0.001
No	166 (48.4)	39 (23.5)	127 (76.5)	
Yes	177 (51.6)	71 (40.1)	106 (59.9)	
Tenure regime of housing				0.000
Owned	774 (71.5)	190 (24.5)	584 (75.5)	
Rented	309 (28.5)	109 (35.3)	200 (64.7)	
Dwelling useful floor area				0.000
Up to 60 m^2^	175 (16.3)	78 (44.6)	97 (55.4)	
61–90 m^2^	415 (38.5)	131 (31.6)	284 (68.4)	
91–120 m^2^	268 (24.9)	62(23.1)	206 (76.9)	
>120 m^2^	219 (20.3)	26 (11.9)	193 (88.1)	
Housing type				0.000
Single-family	240 (22.2)	37 (15.4)	203 (84.6)	
Multi-family	840 (77.8)	261 (31.1)	579 (68.9)	
Overall lighting of housing				0.000
No-little adequate/adequate	419 (39.7)	164 (39.1)	255 (60.9)	
Very-totally adequate	637 (60.3)	129 (20.3)	508 (79.7)	
Dwelling air quality				0.000
Very bad/bad/regular	134 (12.8)	61 (45.5)	73 (54.5)	
Good/very good	916 (87.2)	230 (25.1)	686 (74.9)	
Noise insulation				0.000
No/little insulated	409 (39.1)	153 (37.4)	256 (62.6)	
Properly/very/totally insulated	638 (60.9)	135 (21.2)	503 (78.8)	

* *p* value for the chi-square test of the relationship of the variable with the adequacy of the telework space. *p* < 0.05 implies a statistically significant relationship.

**Table 3:** Aspects of the telework space according to the perception of its adequacy.

Variable	Total N (% col)	No or Little Suitable N (% Row)	Suitable/Very/Totally Suitable N (% Row)	*p* *
General	1121 (100)	308 (27.5)	813 (72.5)	
Persons who telework				0.196
One person	424 (41.5)	108 (25.5)	316 (74.5)	
More than one	597 (58.5)	174 (29.1)	423 (70.9)	
Space for teleworking				0.000
Exclusive use	430 (38.4)	17 (4.0)	430 (96.0)	
Regular multi-purpose	216 (19.3)	51 (23.6)	165 (76.4)	
Temporary multi-purpose	378 (33.8)	167 (44.2)	211 (55.8)	
Roaming space	95 (11.8)	72 (75.8)	23 (24.2)	
Digital resources				0.000
Deficient/insufficient	114 (10.2)	68 (59.6)	46 (40.4)	
Enough	400 (35.9)	131 (32.8)	269 (67.3)	
Good/very good	601 (53.9)	107 (17.8)	809 (72.6)	
Room size				0.000
Not suitable	305 (27.2)	167 (54.8)	138 (45.2)	
Suitable	816 (72.8)	141 (17.3)	675 (82.7)	
Room temperature				0.000
Not suitable	350 (31.2)	151 (43.1)	199 (56.9)	
Suitable	771 (68.8)	308 (27.5)	614 (79.6)	
Daylight entrance				0.000
Not suitable	237 (21.1)	127 (53.6)	110 (46.4)	
Suitable	884 (78.9)	181 (20.5)	703 (79.5)	
Window quality				
Not suitable	649 (57.9)	218 (33.6)	431 (66.4)	0.000
Suitable	472 (42.1)	90 (19.1)	382 (80.9)	
Noise insulation				
Not suitable	671 (59.9)	220 (32.8)	451 (67.2)	0.000
Suitable	450 (40.1)	88 (19.6)	362 (80.4)	
Artificial lighting				0.000
Not suitable	741 (66.1)	248 (33.5)	493 (66.5)	
Suitable	380 (33.9)	60 (15.8)	320 (84.2)	
Furniture				0.000
Not suitable	556 (49.6)	219 (39.4)	337 (60.6)	
Suitable	565 (50.4)	89 (15.8)	476 (84.2)	
Views to the outside				0.000
Not suitable	594 (53.0)	201 (33.8)	393 (66.2)	
Suitable	527 (47.0)	107 (20.3)	420 (79.7)	

* *p* value for the chi-square test of the relationship of the variable with the adequacy of the telework space. *p* < 0.05 implies a statistically significant relationship.

**Table 4:** Telework Space Adequacy Index (TSAI) ranked (1–10).

	Education Level			Tenure Regime		Dwelling Useful Surface (m^2^)				Dwelling Type		Telework Space Adequacy		Usual Work Activity		
	U.G.	G.	P.G.	O	R	0–60	61–90	91–120	>120	H	F	NA/LA	A/VA/TA	P.S.	E.J.	S-E.J.
TSAI (11 telework space aspects)	4.26	5.04	5.23	5.28	4.36	4.22	4.97	5.08	5.67	5.31	4.92	3.42	5.53	5.27	4.73	5.00
TSAIe (5 essential aspects)	5.40	6.17	6.32	6.46	5.32	5.18	6.00	6.25	7.04	6.53	6.01	4.27	6.73	6.45	5.81	6.01
TSAIne (6 non essential aspects)	3.31	4.09	4.32	4.29	3.56	3.42	4.11	4.10	4.52	4.30	4.01	2.72	4.52	4.29	3.83	4.17
Score ranges:		2–3		3–4		4–5		5–6		6–7		7–8				

Table colors represent the TSAI score ranges, as showed caption added above. Acronyms: U.G.: Undergraduate; G: Graduate; P.G.: Postgraduate. O: Owned; R: Rented. H: House; F: Flat. NA: non adequate; LA: little adequate; A: Adequate; VA: very adequate; TA: totally adequate. P.S.: Public Servant; E.J.: Employed job; S-E.J.: Self-employed job. TSAIe: TSAI based on essential categories; TSAIne: TSAI based on non-essential categories.

**Table 5:** Categories and subcategories extracted after graphical analysis of photos.

	Category	Subcategory	
ROOM	Room	Living room	51
		Study	51
		Bedroom	39
		Dining room	19
	Telework space use	Shared fixed space	65
		Shared circumstantial	49
		Roaming space	40
		Exclusive use	24
	Room size	Large space	92
		Small space	83
	Solar control devices		104
	Lighting	Natural	145
		Artificial	48
		Adequate	143
		Not adequate	41
	Views	Buildings	27
		Nature	20
		Own yard	16
TELEWORK AREA	Telework space adequacy	Very/Totally adequate	57
		Adequate	50
		No/little adequate	80
	Digital resource adequacy	Good/very good	84
		Enough	83
		Deficient/Insufficient	16
	Size of available space	Large	79
		Small	105
	Spatial organization	Tidy	120
		Untidy	59
		Loaded	108
		Clear	75
FURNITURE, OTHER ELEMENTS	Ergonomics	Laptop	117
		Computer	70
		Extra fixed PC screen	12
		Ergonomic portable stand	9
		Table_adequate	108
		Table_inadequate	69
		Chair_adequate	73
		Chair_inadequate	72
	Auxiliary furniture	Adequate	61
		Enough	38
		Insufficient	78
	Furniture_shared space		31
	Other elements	Decoration	112
		Radiator	41
		Plants	32
		Children’s objects	28
		Carpets	14
		Organizer panel	13

**Table 6:** Word frequencies found in photo tags from the study participants.

Word Cloud	More Repeated Words	Frequency
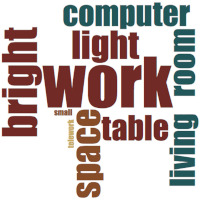	Work	33
	Bright	28
	Light	26
	Space	26
	Table	25
	Computer	25
	Living room	25
	Comfortable	24
	Telework	19
	Small	19

## Data Availability

Data are not available due to ethical reasons.
